# Rescue extracorporeal cardiopulmonary resuscitation in pediatric patients: a nine-year single-center experience in Zagreb, Croatia

**DOI:** 10.3325/cmj.2021.62.146

**Published:** 2021-04

**Authors:** Matija Bakoš, Toni Matić, Filip Rubić, Slobodan Galić, Miran Cvitković, Sandro Dessardo, Darko Anić, Dražen Belina, Milivoj Novak

**Affiliations:** 1 Department of Pediatrics, University Hospital Center Zagreb, Zagreb, Croatia; ^2^Cardiothoracic Surgery Department University Hospital Center Zagreb, Zagreb, Croatia ^3^Department of Cardiac Surgery, University of Zagreb School of Medicine, University Hospital Center Zagreb, Zagreb, Croatia

## Abstract

**Aim:**

To investigate the risk factors and the outcomes of extracorporeal membrane oxygenation (ECMO) in pediatric patients treated at the University Hospital Center Zagreb, the largest center in Croatia providing pediatric ECMO.

**Methods:**

This retrospective study enrolled all the pediatric patients who required E-CPR from 2011 to 2019. Demographic data, cardiac anatomy, ECMO indications, ECMO complications, and neurodevelopmental status at hospital discharge were analyzed.

**Results:**

In the investigated period, E-CPR was used in 16 children, and the overall survival rate was 37.5%. Six patients were in the neonatal age group, 5 in the infant group, and 5 in the “older” group. There was no significant difference between the sexes. Four patients had an out-of-hospital arrest and 12 had an in-hospital arrest. Twelve out of 16 patients experienced renal failure and needed hemodialysis, with 4 out of 6 patients in the survivor group and 8 out of 10 in the non-survivor group. Survivors and non-survivors did not differ in E-CPR duration time, lactate levels before ECMO, time for lactate normalization, and pH levels before and after the start of ECMO.

**Conclusion:**

The similarity of our results to those obtained by other studies indicates that the ECMO program in our hospital should be maintained and improved.

The use of extracorporeal cardiopulmonary resuscitation (E-CPR) is increasing (1). E-CPR is defined as an initiation of extracorporeal membrane oxygenation (ECMO) during active chest compressions. Its main goal is to provide immediate cardiovascular support to patients who do not react to CPR (2) and to lead to survival and a better neurological outcome (3). After administering CPR for more than 30 minutes, survival with conventional CPR measures ranges between 0%-5% (4,5).

The most recent systematic review by the International Liaison Committee on Resuscitation from 2015 recommended that E-CPR should be considered for children with underlying cardiac conditions who have an in-hospital cardiac arrest when appropriate protocols, expertise, and equipment are available (6). According to the Extracorporeal Life Support Organization (ELSO) registry from 2017 (7), more than 60 000 people received extracorporeal life support (ECLS), between 2009 and 2015, with an overall survival rate of 61% (7). Pediatric ECMO experience in Slovenia shows that ECMO programs may be incorporated in smaller hospitals in the region (8-10). The ELSO database includes data on all reported pediatric ECMO runs, including those conducted with E-CPR, and in patients with congenital heart surgery and neonates with diaphragmatic hernia or meconium aspiration syndrome, etc. During the 6-year period, 3005 E-CPR runs were reported, with an overall survival to hospital discharge of 43% (7). A survival rate of 31% was reported by Ergűn et al (11) and in E-CPR patients with severe burn injury (12). The longer the CPR duration time, the lower was the survival to discharge rate. Matos et al reported an E-CPR survival-to-discharge rate of 33% after >35 min of chest compressions (13). Other studies reported that the overall survival rate of pediatric E-CPR cases was growing, with better neurological outcomes than among the patients in the CPR group only (14). Pilar et al found that in 73 pediatric cardiac patients requiring cardiopulmonary resuscitation for >30 min (15), the survival to hospital discharge was 43.8%, with 3/4 of the patients having normal neurological function or mild neurological disability (15). Based on ELSO registry, approximately 10% of all ECMO patients meet brain death criteria (7). One of the biggest single-center studies, involving 184 pediatric ECPR patients (16), showed a successful ECMO weaning in 63% of the patients and the overall survival rate to hospital discharge of 43%. In the same study, the risk factors linked to increased mortality were presupport pH<7.1, mechanical complications, and neurological complications (16). The E-CPR use can involve many complications, not necessarily linked to factors preceding cardiac arrest, such as low cardiac output syndrome or irreversible respiratory failure (17). Furthermore, common complications of ECMO treatment are fluid overload and acute kidney injury (18). Many studies showed renal replacement therapy (RRT) to be negatively associated with survival (15,16,18,19).

This study assessed the risk factors and the outcomes of ECMO in the largest Croatian center providing pediatric E-CPR experience over nine years and compared the survivor and the non-survivor group.

## Materials and methods

This retrospective study enrolled all the children with sudden and refractory cardiac arrest who were treated with E-CPR at the University Hospital Center Zagreb between January 2011 and June 2019. We retrospectively reviewed the records of 16 children and collected demographic data and data on the etiology and location of the arrest, setting of the ECMO run, ECMO type, time of support, and the use of hemodialysis. In Croatia, pediatric ECMO was also instituted in two other hospitals – UHC Rijeka, where it was used for acute respiratory distress syndrome in an 11-month old girl (20), and in UHC Split, where it was used in a patient described below.

The ECMO circuit consisted of coated polyvinyl chloride tubing pack with a CARDIOHELP System and an Oxygenator (Maquet Medical Systems Wayne, NJ, USA). Venoarterial (VA) ECMO was used in all patients. In two patients, a peripheral VA ECMO was instituted through the cannulation of femoral vessels, one patient underwent neck cannulation (right internal jugular vein/right common carotid artery), and other patients underwent central cannulation of the carotid artery and internal jugular vein. None of the patients required the use of inhaled nitric oxygen. The amount of cardiovascular support for hemodynamic instability was calculated with vasoactive inotropic score (VIS). The score was dichotomized into normal (VIS<20) and high (VIS>20). The decision to start E-CPR was not standardized and was made by the patient’s primary bedside physician. We defined the E-CPR duration as the time between the start of the chest compressions and the ECMO flow initiation. Laboratory data before and after ECMO cannulation were also obtained. In the pediatric intensive care unit (PICU), standard heart ultrasound was performed twice daily. Therapeutic hypothermia after CPR and during ECMO was not used, although hyperthermia was aggressively treated. Survival was defined as a survival to hospital discharge, or, where specified, as a survival to ECMO decannulation. Children who underwent E-CPR were neurologically evaluated by radiologic imaging: computed tomography, ultrasonography, and magnetic resonance. Neurological outcome was assessed by a pediatric neurologist using pediatric cerebral performance score. Concerning cardiac surgery, none of the patients were re-operated. The study was approved by the University Hospital Center Zagreb Institutional Review Board (02/21 AG).

### Inclusion and exclusioncriteria

The criteria for choosing the patients for rescue ECMO were in- or out-of-hospital cardiac arrest, associated or not with congenital heart surgery, in which CPR (including bystander) started within 10 min after the arrest. We included the patients with CHD both before and after cardiac surgery. All the patients were aged from 0 to 18 years. CPR was defined as a procedure that requires chest compressions, not only rescue breaths. The ECMO team was activated by the pediatrician in charge of the resuscitation. The study did not include a neonate with congenital diaphragmatic hernia in whom CPR had started and ECMO had been indicated but not deployed because of technical problems.

### Statistical analysis

Descriptive statistics were summarized as counts and percentages for categorical variables and as median and interquartile range (IQR) for continuous variables. The Fisher exact, Mann-Whitney, and Pearson χ^2^ test were used to compare categorical and numerical variables, while some data were analyzed descriptively. A *P* value <0.05 was considered significant. The analyses were performed with the SPSS, version 20.0 (IBM Corp., Armonk, NY, USA).

## Results

From January 2011 to June 2019, ECMO support during CPR was initiated in 16 patients. Four of them had an OHCA and 12 had an IHCA. Six patients were neonates, 5 were infants, and 5 belonged to the “older” group, encompassing all the children up to 18 years. The average age for starting CPR was 5 months (IQR; 1-67 months). Six patients were female (37.5%). The average weight was 4.7 kg (IQR 3.225-14.58 kg), height 57 cm (IQR 52-95.5 cm), and body surface area 0.275 m^2^ (IQR 0.22-0.63 m^2^). Cardiorespiratory arrest was caused by circulatory insufficiency in 15 out of 16 patients (94%) and by a respiratory cause in one patient, a neonate with diaphragmatic hernia. In this patient, CPR lasted 160 minutes, and ECMO lasted only 8 hours, due to the unfavorable disease course.

In 63% of patients, circulatory insufficiency was caused by a congenital heart defect (CHD), while the other causes included myocarditis, cardiomyopathies, and sodium valproate intoxication. ECMO treatment was performed after cardiac surgery in patients with tetralogy of Fallot (one patient), coarctation of the aorta with ventricular septal defect, patent foramen ovale, and patent arterial duct (three patients), total anomalous pulmonary venous return (TAPVR) – infradiaphragmatic type, univentricular heart, and transposition of the great arteries (one patient), complete atrioventricular canal (one patient), and TAPVR as a part of a heterotaxy syndrome and with double inlet left ventricle and pulmonary atresia (one patient). In conclusion, five patients had RACHS score 2, two patients RACHS had score 3, one patient had RACHS score 4, and one patient had RACHS score 5. One patient had severe aortic valve stenosis but cardiac surgery was not performed. Male and female groups did not significantly differ in any of the parameters (Table 1). Two patients had associated anomalies (conatal hypothyreosis and hetorotaxy syndrome) and neither of them survived (Table 2). Four E-CPR patients underwent bystander CPR in out-of-hospital settings (OHCA). Three were successfully weaned from ECMO, and 2 of them survived, both without neurological consequences. Four out of 12 IHCA patients survived. Seven out of 16 (43.7%) patients survived to ECMO decannulation and 6 out of 16 (37.5%) survived to discharge. In the OHCA group, 3 out of 4 patients survived to ECMO decannulation and 2 out of 4 survived to hospital discharge, and brain death was confirmed in one patient. Survivors had longer CPR duration times (94 [45-180] min) compared with non-survivors (29 [5-180] min). They also had longer PICU stay (27 vs 15 days) and in-hospital stay (79 vs 23 days). Hemodialysis was used in 12 patients, but without significant difference between survivors and non-survivors. ECMO duration was somewhat shorter in the survivor group (138.5 vs 169 hours; *P* > 0.05), and the initial flow was somewhat higher (2186.5 vs 1725 ml/m^2^; *P* > 0.05). No significant difference in lactate levels was found before or during lactate normalization after starting ECMO. Moreover, survivors and non-survivors did not differ in pH, BE, and pCO_2_ levels before and after starting the ECMO procedure (Table 3). The most common ECMO complications were acute bleeding (intracranial, intraabdominal, intrathoracic) in 11 patients (3 in the survivor group) and signs of sepsis in 10 patients (2 in the survivor group), but the difference between survivors and non-survivors in both cases was not significant (Table 3). In the last 3 patients included in the study, the ECMO run was started after OHCA. The last patient who survived E-CPR was hospitalized in the UHC Split, where ECMO run was started and was immediately transferred to our hospital early in 2019. He is now a 16-year-old diagnosed with restrictive cardiomyopathy and bridged to left ventricular assist device 21 days after VA ECMO support. Two months later, heart transplantation was successfully performed. Subsequently, a mutation in troponin I (TNNI3) was found.

**Table 1:** Differences between male and female pediatric patients who underwent extracorporeal membrane oxygenation (ECMO) from 2011 to 2019*

	Male		Female		P
	**yes**	**no**	**yes**	**no**	
**Associated malformations**	0	8	2	4	0.165
**Neurological damage**	3	5	5	1	0.121
**Hemodialysis**	6	4	6	0	0.115
**Former cardiac surgery**	1	5	2	1	0.226
**Lactate normalization in 24 hours of ECMO run**	5	2	3	2	0.576
Base excess **before starting ECMO<-14**	5	3	2	2	0.576
**Lactate level before starting ECMO>10 mmol/L**	8	0	2	2	0.091

**Table 2:** Differences between survivors and non-survivors after extracorporeal membrane oxygenation (ECMO). The data are expressed as counts (%) unless otherwise indicated*

	Survivors	Non-survivors	P value
**Patients' characteristics**			
**Associated malformations**	0	2 (28)	0.396
**Neurological damage**	0	8 (89)	0.003
**Hemodialysis because of renal failure**	4 (67)	8 (80)	0.489
**Bypass before arrest**	1 (17)	6 (60)	0.121
**Pediatric intensive care unit stay (days), median (IQR)**	27 (14-104)	15 (1-26)	0.022
**Hospital stay (days), median (IQR)**	79 (37-220)	23.5 (1-121)	0.019
**Advanced life support, duration before ECMO (minutes), median (IQR)**	94 (45-180)	29 (5-180)	0.181
**Location of ECPR**			
**Pediatric intensive care unit**	3	3	0.213
**Operating theater**	1	6	
**Other (hospital + emergency department)**	2	1	
**ECMO type**			
**VV ECMO**	0	1	0.625
**VA ECMO**	6	9	
**ECMO characteristics**			
**Normalization of lactate level in 24 hours of ECMO run**	4 (100)	4 (50)	0.141
**Successfully weaned from ECMO**	6 (100)	1 (10)	0.001
**ECMO duration, median (IQR)**	138.5 (67-504)	169 (7-578)	0.0968
**Initial flow (ml/m2), median (IQR)**	2186.5 (1550-2500)	1725 (610-2650)	0.518

*Abbreviations: ECPR – ECMO cardiopulmonary resuscitation, VV – veno venous; VA – veno arterial.

**Table 3:** Patients' characteristics before and after extracorporeal membrane oxygenation (ECMO) treatment. The data are expressed as mean (interquartile range) unless otherwise indicated

	Survivors		Non-survivors		P
**Blood gas analysis before ECMO**					
**pH**	7.12 (6.95-7.425)		6.99 (6.99-7.26)		0.464
**pCO_2_ (kPa)**	5.7 (4.8-9.75)		8.4 (3.7-8.8)		0.807
**pO_2_ (kPa)**	11.1 (3.9-17.1)		7.9 (2.7-15.1)		0.570
**base excess**	-9.1 (-23.5-0.8)		-20.2 (-22.5-(-10.1))		0.465
**Bleeding on ECMO**	yes	no	yes	no	0.210
	3	3	8	2	
**Signs of sepsis before ECMO decannulation**	yes	no	yes	no	0.062
	2	4	8	2	
**Vasoactive inotropic score before ECMO (unknown for 4 patients)**	<20	>20	<20	>20	0.453
	3	2	2	5	

## Discussion

In our study, survival to hospital discharge after E-CPR was 37.5%. The first pediatric ECMO in Croatia was performed in November 2009, and up to early 2020 there were 48 ECMO runs. Since the first successful case, more than a year passed before the second ECMO run, which was our first pediatric ECMO after providing CPR. In the last decade, an increasing trend of pediatric E-CPR use was observed, with three patients from 2011 to 2013 and nine patients from 2017 to 2019. Barbaro et al (7) reported that from 2009 to 2015 neonatal E-CPR use increased by 35% (from 108 to 146 annual cases), while pediatric E-CPR use increased by 67% (from 221 to 369 cases per year). According to the ELSO registry report, in the last decade the use of pediatric ECMO has increased 10-fold, while hospital survival rate has not considerably changed (39% in 2004 and 41%) (3,7). Similar hospital survival rates were also observed in other studies (21,22). Most recently, Torres-Andres et al have reported E-CPR survival to hospital discharge of 65.5% and survival at the end of the follow-up period of 62.1% (3). Median follow-up time was 3 years (IQR 1.5-4.5) (3). In our study, 75% of the patients had an IHCA, with the hospital survival rate of 33%. The remaining 25% had an OHCA, with a hospital survival rate of 50%, which is a rate higher than those reported in other studies (3,7,21,22). Interestingly, the last three patients had E-CPR after OHCA.

Unlike other studies, we found no significant difference between survivors and non-survivors in E-CPR duration time, lactate levels before ECMO, time for lactate normalization, and pH levels (22-24). A potential explanation could be the small sample size in our study. The reason why E-CPR duration did not significantly affect survival could be that two patients in the survivor group had E-CPR duration time of 150 and 180 minutes. Both of them were male infants who arrested in the postoperative period after cardiac surgery. Since the patients had an IHCA, CPR was started immediately. The 180-minute resuscitation patient had intervals of ROSC shorter than 5 min for a number of times after 20 minutes of CPR, but the arrest continuously reoccurred. Both patients were successfully decannulated and discharged from hospital with no neurological impairment. On the other hand, two infants with the sternum left open arrested soon after the PICU admission after cardiac repair, so the ECMO team was immediately available to perform ECMO. The CPR time was short (five and seven minutes), but the outcome was still undesirable. All of the six surviving patients had the CPR time longer than 45 minutes. Three of them had the CPR time longer than two hours. All of them survived without neurological impairment. It is a subject to discussion whether therapeutic hypothermia can improve the neurological outcome after CPR (15). None of our 16 E-CPR patients underwent therapeutic hypothermia, but hyperthermia was agressively treated. In a study by Alsoufi, seven patients survived after more than one hour of E-CPR, without any gross neurological deficit (2). Recent studies have shown that children can survive after >30 minutes of in-hospital CPR (13,25). Pilar et al found that E-CPR longer than 50 minutes predicted the fatal outcome. They, however, described three patients who survived very long CPR episodes (118, 134, and 139 min) with mild neurological deficits, similar to the results of our study (15). This findings indicate that survival without neurological impairment can be expected even in CPR lasting more than one hour.

The small sample size prevented us from investigating the complexity of cardiac lesions and their effect on mortality and morbidity. Other studies reported the effect of the type and complexity of congenital heart defects on ECMO outcomes. The mortality rate increased with a higher RACHS score (26). As we are a small center, the largest proportion of pediatric patients who undergo cardiac operations have a RACHS score of 4 and lower. Only one patient with a score 5 was treated with ECMO after E-CPR due to pulmonary hypertension and low cardiac output syndrome after truncus arteriosus repair. Inhaled nitric oxygen was not used during ECMO, only before ECMO was started. The infant born with 720 g had an unfavorable outcome after 561 hours of ECMO due to hemodynamic instability, the need for RRT, and invasive aspergillosis infection. Harmes et al (27) published the largest study so far including only patients with ECMO support after truncus arteriosus repair. They analyzed 245 patients (mortality rate of 62.4%) and found that independently associated risks factors for mortality were lower weight, longer ECMO duration, the need for RRT, and infection on ECMO (27,28).

Longer CPR time in our study can be partly explained by a lack of local guidelines for activating the ECMO team. In all of the cases, the ECMO team was activated by the pediatrician in charge of resuscitation. A particular problem are the night shifts, when only one pediatrician is in charge, very often directly involved in CPR. Problems are also present during the transport from the emergency department to PICU, where special attention needs to be placed on the compression depth and rate (29). In the single-center experience of pediatric E-CPR by Pilar et al, CPRs were longer than 30 minutes. They found a strong difference between survivors and non-survivors in ECMO team response (15). Furthermore, survivors had a shorter median interval to ECMO team activation as well as shorter CPR and ECMO duration (15). Other authors reported applying ECPR within 5 minutes of the arrest, after having excluded the patients with fatal comorbidities (16). The outcomes in our center can be improved by creating local guidelines for activating the ECMO team.

Twelve out of 16 patients experienced renal failure and needed hemodialysis, with 4 out of 6 patients in the survivor group and 8 out of 10 in the non-survivor group. The remaining two patients in the non-survivor group did not undergo hemodialysis because the ECMO run lasted for only 7 minutes in one patient and 47 minutes in the other. This and the small sample size are probably the reasons why the difference between survivors and no survivors in the need for hemodialysis did not reach significance. Consequently, the results are different from other studies, where both kidney injury and renal replacement therapy were independent predictors of mortality (16,30).

Regarding the associated malformations, patients with trisomy 21 make up to 20% of syndromic patients needing ECMO treatment (11). Furthermore, studies show that the presence of Down and DiGeorge syndrome does not affect survival after ECMO (24,31). Conversely, Ergun et al found that the presence of a syndrome increased mortality (11). In our study, two of our 16 patients (12.5%) had associated medical conditions, and both were in the non-survivor group.

The use of pediatric ECMO in our hospital is increasing. A total of 16 E-CPR runs were performed in last nine years, but the number was disproportional during the years. In the first four years, 5 ECMO runs were performed after CPR, and 11 in the last four years. The number is still too low to allow making a definite conclusion, but an increased number of ECMO runs after CPR in the last few years encourages us to develop our local guidelines. Finally, our overall results are similar to other published studies, which is a reason to maintain the ECMO program in our hospital with an aim to improve the outcome of every new patient.

This study has some important limitations. First, it is a single-center study with a relatively small sample size, which is understandable as cardiac arrest in children is not as common as in the adult population. Furthermore, as the number of patients is limited, the number of events is also low, meaning that the outcome is affected by several factors, which cannot be included in a multi-variable analysis. Second, the absence of some data in patients' histories due to the retrospective nature of the study can be an important confounder. We hope that a prospective database, which is currently being instituted, will help us more accurately analyze prognostic factors. Finally, the neurodevelopmental outcome was assessed with a simple scoring system, without uniform criteria on imaging studies.

In conclusion, E-CPR should be used as a rescue treatment for in- and out-of-hospital pediatric cardiac arrest refractory to initial procedures. If not on standby already, ECMO team should be immediately activated when an IHCA occurs. Although derived from a small sample, the results of OHCA patients are encouraging for the implementation of an ECMO protocol in as many regional hospital centers as possible.
